# New York State Veterinary College, Cornell University

**Published:** 1900-07

**Authors:** 


### NEW YORK STATE VETERINARY COLLEGE, CORNELL UNIVERSITY.

At the annual commencement exercises held at Ithaca, N. Y., June 21st, the degree of D.V.M. was conferred on the following: Clarence Lyon Barnes, Lockport, N. Y.; John W. Corrigan, Owego, N. Y.; Grant Sherman Hopkins, Westport, N. Y.; Charles H. Jewell, Staterville Springs, N. Y.; Louis Greene Juliand, New York; William J. Mitchell, Ithaca, N. Y.; Garry T. Stone, Binghamton, N. Y.

The Horace K. White prizes of $15 and $10 to the most meritorious students in the graduating class were awarded to Clarence L. Barnes and Garry T. Stone.

The theses submitted by the candidates for graduation were all founded on original work on the subjects selected, as follows:

C. L. Barnes, Laryngeal Hemiplegia and Roaring; J. W. Corrigan, Melanosis and Melanoma; G. S. Hopkins, the Brachial Plexus in Monodactyle and Polydactyle; C. H. Jewell, Ovariotomy in Domestic Animals; L. Juliand, Aloin as a Purgative; W. J. Mitchell, Bacteria in the Female Generative Organs; Garry T. Stone, Tuberculin and Its Uses in Diagnosis.

